# Detailed role of mesenchymal stem cell (MSC)-derived exosome therapy in cardiac diseases

**DOI:** 10.17179/excli2023-6538

**Published:** 2024-03-25

**Authors:** Ali Hassanzadeh, Navid Shomali, Amin Kamrani, Hadi Nasiri, Javad Ahmadian Heris, Maryam Pashaiasl, Mohammadreza Sadeghi, Shahram Sadeghvand, Zahra Valedkarimi, Morteza Akbari

**Affiliations:** 1 Department of Applied Cell Sciences, School of Advanced Technologies in Medicine, Tehran University of Medical Sciences, Tehran, Iran; 2 Department of Immunology, Faculty of Medicine, Tabriz University of Medical Sciences, Tabriz, Iran; 3 Immunology Research Center, Tabriz University of Medical Sciences, Tabriz, Iran; 4 Department of Allergy and Clinical Immunology, Pediatric Hospital, Tabriz University of Medical Sciences, Tabriz, Iran; 5 Department of Anatomical Sciences, Faculty of Medicine, Tabriz University of Medical Sciences, Tabriz, Iran; 6 Women’s Reproductive Health Research Center, Tabriz University of Medical Sciences, P.O. Box 51376563833, Tabriz, Iran; 7 Department of Molecular Medicine, Tabriz University of Medical Sciences, Tabriz, Iran; 8 Pediatrics Health Research Center, Tabriz University of Medical Sciences, Tabriz, Iran; 9 Department of Medical Biotechnology, Faculty of Advanced Medical Sciences, Tabriz University of Medical Sciences, Tabriz, Iran

**Keywords:** mesenchymal stem cell (MSC), exosome, cardiac diseases, treatment, regeneration

## Abstract

Coronary heart disease (CHD) continues to be the leading cause of morbidity and mortality. There are numerous therapeutic reperfusion methods, including thrombolytic therapy, primary percutaneous coronary intervention, and anti-remodeling drugs like angiotensin-converting enzyme inhibitors and beta-blockers. Despite this, there is no pharmacological treatment that can effectively stop cardiomyocyte death brought on by myocardial ischemia/reperfusion (I/R) injury. For the purpose of regenerating cardiac tissue, mesenchymal stem cell (MSC) therapy has recently gained more attention. The pleiotropic effects of MSCs are instead arbitrated by the secretion of soluble paracrine factors and are unrelated to their capacity for differentiation. One of these paracrine mediators is the extracellular vesicle known as an exosome. Exosomes deliver useful cargo to recipient cells from MSCs, including peptides, proteins, cytokines, lipids, miRNA, and mRNA molecules. Exosomes take part in intercellular communication processes and help tissues and organs that have been injured or are ill heal. Exosomes alone were found to be the cause of MSCs' therapeutic effects in a variety of animal models, according to studies. Here, we have focused on the recent development in the therapeutic capabilities of exosomal MSCs in cardiac diseases.

## Introduction

Cardiovascular diseases are the leading cause of morbidity and mortality worldwide, particularly coronary heart disease (CHD) (CDC, 2011[16]). Acute myocardial infarction (MI) is the main reason for death in all CHDs. A substantial medical, social, and financial burden results from subsequent complications like heart failure (HF) (Reddy et al., 2015[103]). In addition to anti-remodeling drugs like angiotensin-converting enzyme inhibitors and beta-blockers, a wide range of curative reperfusion plans are accessible, including thrombolytic therapy and primary percutaneous coronary intervention (Rich, 2006[107]). However, no pharmacological treatment can effectively stop cardiomyocyte destruction brought on by myocardial ischemia/reperfusion (I/R) (Kalogeris et al., 2016[55]).

Additionally, cardiac fibrosis, myocardial remodeling, cardiac arrhythmia, and ultimately heart failure could all be influenced by this I/R injury. Heart transplantation or ongoing left ventricular (LV) support are the only therapies for treating heart failure at its most advanced stage (Mangini et al., 2015[81]). Therefore, there is great interest in and demand for novel remedies for post-MI LV remodeling and dysfunction.

Due to their involvement in numerous facets of cardiac biology and disease, exosomes significantly impact cardiac health (Yao et al., 2021[133]). The importance of exosomes in cardiac health is highlighted in the following key points. Exosomes help the heart's intercellular communication by carrying biological information between cells. Cells can communicate and coordinate their functions thanks to the molecules they transfer, which also have proteins, genetic material (like RNA), and other molecules (Harrell et al., 2020[46]: Lu et al., 2018[70]). Exosomes are essential for heart tissue regeneration and repair. They may contain regenerative substances that encourage cardiac progenitor cells to multiply and differentiate, aiding in repairing damaged cardiac tissue after trauma or illness (Liu et al., 2022[65]). Numerous cell types, including stem cells and cardiomyocytes, have been shown to produce exosomes that have cardioprotective properties (Vrijsen et al., 2010[122]). They can lower heart-related oxidative stress, inflammatory response, and cell death, maintaining cardiac function and enhancing recovery from cardiac conditions (Cosme et al., 2013[24]). Importantly, exosomes from mesenchymal stromal cells (MSCs) have a promising future in regenerative medicine (Lai et al., 2011[60]; Nasser et al., 2021[89]). They are a strong candidate for cardiac regeneration and repair due to their potential trophic and immunomodulatory effects (Han et al., 2019[45]). Exosomes produced by MSCs are considered a viable alternative to cell-based therapies because numerous studies have shown that they mimic their parent cells' anti-inflammatory, anti-apoptotic, pro-angiogenic, and anti-fibrotic possessions (Deng et al., 2019[29]; Yue et al., 2022[135]). They are desirable in regenerative medicine due to their superior immune tolerance, stability, and lower tumorigenic risk than their parent stem cells (Ahmed and Al-Massri, 2022[3]). Therefore, the potential for using MSC-derived exosomes for cardiac renewal and repair has been assessed.

This article offers an organized framework for compiling and presenting data on the therapeutic capability of MSCs-exosomes in cardiac diseases. 

## Overview of Cardiac Conditions

Heart failure and myocardial infarction are common cardiac conditions that seriously impact people's health and well-being. A compromised heart's ability to pump blood efficiently may result in heart failure (HF) (Groenewegen et al., 2020[42]). Several underlying conditions, including coronary artery disease, hypertension, or damaged heart valves, may bring it on. Breathing difficulty, exhaustion, fluid retention, and a decreased capacity for exercise are all signs of heart failure (Bader et al., 2021[8], Lopaschuk et al., 2021[68]). It is known as a MI when the blood supply to a portion of the heart muscle is cut off, typically due to a blood clot in the coronary arteries (Reed et al., 2017[104]). The heart muscle cells die from this blockage, causing chest pain, breathlessness, and potentially fatal complications. In addition, these symptoms characterize coronary artery disease (CAD), which restricts or blocks the arteries that carry blood to the heart muscle (Frangogiannis, 2011[37]; Saleh and Ambrose, 2018[108]). It is typically brought on by plaque development in the artery walls, which comprises cholesterol, fat, and other substances (Okrainec et al., 2004[94]). Heart attack, heart failure, or angina are all possible CAD outcomes. Arrhythmias, abnormal heartbeats, can also impair the heart's typical ability to pump blood (Libby and Theroux, 2005[64]). They might show up as tachycardia, bradycardia, or an irregular heartbeat. Symptoms of arrhythmias include palpitations, lightheadedness, fainting, and, in extreme cases, cardiac arrest (Malakar et al., 2019[79]). Also, valvular heart disease describes illnesses that affect the heart valves and compromise their ability to control blood flow properly. Fatigue, breathlessness, chest pain, and fluid retention are signs and symptoms of valvular heart disease (Aluru et al., 2022[5]).

Additionally, a group of cardiomyopathies affects the heart muscle, resulting in structural and functional abnormalities (Crisafulli et al., 2020[25]). The heart may enlarge, thicken, or stiffen due to these conditions, which can be genetic or acquired (Tesson et al., 2019[119]). Cardiomyopathies can bring on heart failure, arrhythmias, and other complications. For the best possible patient outcomes, these disorders must be appropriately assessed, diagnosed, and managed medically (Neisius et al., 2019[90]).

## Mesenchymal Stem Cell in Cardiac Regeneration

Based on *in vivo* reports, MSCs therapy has attracted increasing attention in cardiac regeneration, more importantly, MI (Table 1(Tab. 1); References in Table 1: Berry et al., 2006[10]; Dai et al., 2005[26]; de Macedo Braga et al., 2008[28]; Fazel et al., 2005[35]; Grauss et al., 2008[41]; Gyöngyösi et al., 2008[44]; Imanishi et al., 2008[53]; Kudo et al., 2003[58]; Li et al., 2007[63]; Makkar et al., 2005[78]; Mangi et al., 2003[80]; Nagaya et al., 2004[86]; Nakamura et al., 2007[88]; Noiseux et al., 2006[93]; Perin et al., 2008[97]; Price et al., 2006[98]; Quevedo et al., 2009[101]; Schuleri et al., 2009[110]; Shake et al., 2002[111]; Shiota et al., 2007[113]; Silva et al., 2005[114]). MSCs can be differentiated into cardiomyocytes, endothelial cells, and vascular smooth muscle cells by the contribution of various paracrine effectors, finally promoting cardiac repair and regeneration (Taylor and Robertson, 2009[118]). MSCs can arouse the production of manifold growth factors, replace injured cells, and create an environment to favor endogenous cardiac rehabilitation. 5-Azacytidine (5-AZA), a well-known inhibitor of DNA methylation, is a chemical ingredient that can induce BM-MSCs differentiation into cardiomyocytes, as shown in rodents (Jia et al., 2020[54]; Makino et al., 1999[77]). Moreover, the supportive impacts of the miR-1a overexpressing on BM-MSCs differentiation into cardiac cells has recently been suggested (Zhao et al., 2016[138]). Other studies have demonstrated that IL-1β contributes to the pathogenesis, development, and function of cardiomyocytes in the injured heart and can induce neovascularization post-MI (Guo et al., 2018[43]). Recently, BM-MSCs therapy improved cardiac function in rodents with MI by stimulating cardiac endothelial cell (CEC) migration to the infarcted border area primarily through CXCL12/CXCR4 axis (Lu et al., 2019[69]). Recently, in patients with compensated HF, intravenous (IV) administration of allogeneic UC-MSCs and BM-MSCs led to a remarkable increase in the expression of hepatocyte growth factor (HGF), finally causing myogenesis and suppression of inflammation (Bartolucci et al., 2017[9]). Also, IV administration of UC-MSCs promoted left ventricular function and life quality in HF patients (Bartolucci et al., 2017[9]). In 6 patients with MI, autologous MSCs therapy also caused concordant amelioration in regional activity, tissue perfusion, and, eventually, fibrotic burden (Karantalis et al., 2014[57]). 

## Exosomes in Cardiac Regeneration

### Explanation of exosome function in tissue repair

Exosomes are essential for intercellular communication and the delivery of vital biological signals to recipient cells engaged in the healing process, which is critical for tissue repair (Huang et al., 2021[50]). Exosomes carry a cargo of proteins, nucleic acids (like RNA), lipids, and other molecules necessary for cellular contact and tissue repair (Bjørge et al., 2018[11]). Growth factors, cytokines, enzymes, and genetic material that control various cellular processes can all be found in these cargos. Different cell types, including stem cells, immune cells, and damaged or injured cells, secrete exosomes (Newton et al., 2017[91]). These cells produce exosomes reacting to cellular stress, injury, or particular signaling cues that target unique bioactive molecules. Depending on the specific mechanisms, exosomes can be selectively taken up by around or distant cells (Fan et al., 2022[32]; Lai et al., 2010[59]). The transfer of their cargo into the recipient cells is made possible by their ability to either fuse with or bind to the cell membrane of the target cells.

Exosomes carry molecules that activate regenerative signaling pathways in recipient cells as part of their cargo (Fang et al., 2019[34]). Exosomes, for example, can stimulate signaling pathways that promote cell migration, proliferation, and differentiation. Exosomes can control inflammation in harmed tissue by modifying immune reactions (Su et al., 2021[116]; Toh et al., 2018[121]). They may transport molecules that control excessive inflammation and encourage a tissue's ability to regenerate. Exosomes can also promote angiogenesis, the growth of new blood vessels, in the injured tissue (Qin et al., 2016[100]). Transferring pro-angiogenic components to endothelial cells can encourage their growth, migration, and the development of new blood vessels, which are essential for furnishing the regenerating tissue with oxygen and nutrients (Manuel et al., 2017[82]).

Additionally, they affect the remodeling of the extracellular matrix (ECM), the framework that supports cells and thus aids in tissue repair. Exosomes can transport enzymes that break down or alter the ECM, making eliminating harmed matrix elements easier and depositing fresh ECM proteins needed for tissue regeneration (Chen et al., 2019[19]; Wen et al., 2021[128]). Exosomes play various roles in tissue repair, including transporting bioactive molecules, delivering those molecules to recipient cells, activating regenerative signaling pathways, reducing the inflammatory response, promoting angiogenesis, and participating in ECM remodeling (Moghadasi et al., 2021[85]). These procedures help damaged tissues heal and regenerate in various situations, such as wound healing, cardiac repair, and tissue regeneration following disease or injury.

### Detailed role of exosomes in cardiac regeneration

**Figure 1:**
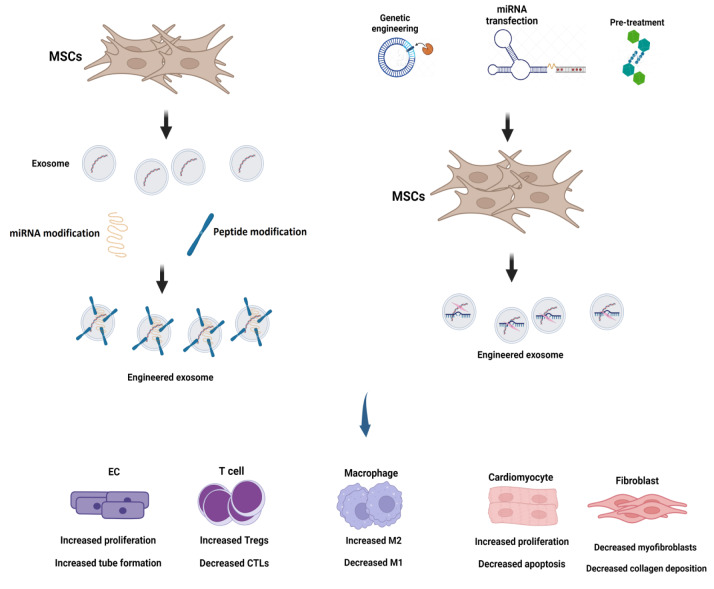
Engineered mesenchymal stem cell (MSC)-derived exosome in cardiac regeneration (created by BioRender).

Exosomes produced by different cell types, including stem cells, cardiac progenitor cells, and mesenchymal stem cells, function as paracrine signaling messengers. They deliver bioactive molecules, such as growth factors, cytokines, and miRNAs, to recipient cells in the damaged cardiac tissue. Exosomes have the potential to aid in the injured heart's angiogenesis and feed endothelial cells pro-angiogenic substances like vascular endothelial growth factor (VEGF), which encourages the growth of new blood vessels (Wang et al., 2016[125]). Improved blood flow makes reaching the regenerating tissue easier for nutrients and oxygen. Cardiomyocytes, the muscle cells liable for the heart's contraction, can survive and increase with the help of exosomes (Garcia et al., 2015[39]). They distribute substances that promote the proliferation of cardiomyocytes and prevent cell death (apoptosis). MiRNAs like miR-133 and miR-210, which have been demonstrated to improve cardiomyocyte survival and proliferation, can be one of these factors (Cervio et al., 2015[17]; Ma et al., 2018[76]). Exosomes can modify cardiac fibrosis, a condition marked by excessive creation of extracellular matrix proteins that results in scar tissue formation (Ranjan et al., 2019[102]). They can transfer molecules that control the fibroblast's activity, which broadly participate in ECM generation and performance (Tikhomirov et al., 2020[120]). Exosomes can attenuate fibrosis and encourage tissue regeneration by altering the equilibrium between collagen synthesis and degradation. Exosomes support the injured cardiac tissue's immune system to be modulated. They may contain anti-inflammatory substances like transforming growth factor-beta (TGF-β) or interleukin-10 (IL-10), which reduce extravagant inflammation and encourage regeneration (Wen et al., 2021[128]). This immunomodulatory impact contributes to developing an advantageous microenvironment for cardiac regeneration. There is growing evidence that exosomes play a role in ECM remodeling, essential for the structural integrity and practical restoration of the injured cardiac tissue (Cao et al., 2021[14]). They deliver enzymes and matrix remodeling elements that aid in breaking down harmed ECM elements and synthesizing fresh ECM proteins, promoting tissue remodeling and regeneration. Exosomes can also influence several cellular functions, including cell migration, differentiation, and metabolism, which are crucial for cardiac regeneration (Ranjan et al., 2019[102]). They have the ability to transfer regulatory molecules, such as proteins and miRNAs, to control these processes in recipient cells and enhance their capacity for regeneration (Emanueli et al., 2016[31]; Nasser et al., 2021[89]). The role of exosomes in cardiac regeneration highlights their potential as therapeutic agents for heart diseases. To improve outcomes in conditions like myocardial infarction, heart failure, and other cardiac pathologies, scientists are working to develop exosome-based therapies that increase cardiac tissue repair (Figure 1(Fig. 1)).

## Therapeutic Application of MSC-Derived Exosome

Due to their inherent capacity to transport biomolecules between cells, exosomes have drawn considerable interest as a potential means of delivering therapeutic cargo. Exosomes are helpful delivery agents because of their small size, stability, biocompatibility, and capacity to target particular cell types (Table 2(Tab. 2); Reference in Table 2: Hassanzadeh et al., 2021[48]; Moghadasi et al., 2021[85]) (Xiao et al., 2018[129]; Zhu et al., 2018[139]).

Exosomes can be created artificially or loaded with therapeutic cargo (Das et al., 2018[27]). Drugs, growth factors, siRNA, miRNA, and gene-editing tools are examples of the cargo that may be present (Peng et al., 2020[96]). Incubation, electroporation, sonication, genetic modification of the parent cells that make exosomes, and other techniques can all be used to load (Lai et al., 2020[61]). Exosomes can be altered to improve their targeted abilities. Specific targeting ligands can be added, enabling exosomes to connect to receptors on target cells selectively; this can be accomplished by altering the surface proteins of exosomes or their membrane structure. This targeting strategy permits the precise delivery of therapeutic cargo to particular cell types, increasing efficacy and reducing off-target possessions (Feng et al., 2014[36]). Exosomes interact with other cells within the body and deliver therapeutic cargo directly to recipient cells (Luo et al., 2017[73]). Beyond the therapeutic benefits of the transferred cargo itself, the transmitted cargo can modify the recipient cells' gene expression, signaling pathways, or cellular behavior. In addition to promoting tissue repair and improving therapeutic results, this communication can aid in controlling cellular processes.

### MSC-exosome in HF

MSCs-exosomes have cardioprotective properties. Interleukin (IL)-1, IL-4, IL-6, tumor necrosis factor (TNF), and brain natriuretic peptide (BNP) levels were decreased by exosome therapy in animal models of heart failure (HF) (Ren et al., 2023[105]).

In a mouse model of pressure-overload heart failure, Nakamura et al. examined the cardioprotective effects of intravenously injected adiponectin-induced MSCs-exosome (Nakamura et al., 2020[87]). Exosomes, extracellular vesicles with endosome origins produced by the injected MSCs, are responsible for their function. Because T-cadherin is a special glycosylphosphatidylinositol-anchored cadherin on MSCs, adiponectin stimulated exosome biogenesis and secretion by binding to it (Nakamura et al., 2020[87]). Promising findings were achieved from a study on the prevention of heart failure (HF) in MI rats using BMMSCs-exosomes expressing microRNA-30e (miR-30e) (Pu et al., 2021[99]). Exosome overexpressing miR-30e was administered to rats to treat the pathological injury, cardiomyocyte apoptosis, and fibrosis in rat myocardial tissues.

Additionally, miR-30e negatively regulated LOX1 expression, which was overexpressed in the MI rats, but additional exosome treatment restrained LOX1 expression. Further, exosome overexpressing miR-30e reduced NF-κB p65/Caspase-9 signaling in the myocardial tissues of MI rats, reducing cardiomyocyte apoptosis and fibrosis (Pu et al., 2021[99]). By blocking the NF-κB signaling pathway, the miR-129-5p enriched MSC-Exos injection reduced ventricular dysfunction and reduced oxidative stress, apoptosis, inflammation, and fibrosis in cardiomyocytes in mice with HF (Yan et al., 2022[132]) - exosomal miR-129-5p from MSCs guards against heart failure by focusing on TRAF3 and the subsequent NF-κB signaling. This regulatory axis could be a potential therapeutic target for HF (Yan et al., 2022[132]). Similarly, exosomal miR-1246 released from human UCMSCs reduced hypoxia-induced myocardial tissue damage by targeting PRSS23 and preventing the activation of the Snail/alpha-smooth muscle actin signaling. By targeting PRSS23 and encouraging angiogenesis, exosomal miR-1246 from hucMSCs does guard the heart against failure (Wang et al., 2021[126]).

### MSC-exosome in MI

MSCs-exosomes have a cardioprotective effect in rats with I/R injury, as shown by a significant reduction in I/R-induced myocardial infarction and a drop in the serum levels of cardiac troponin I (cTnI), lactate dehydrogenase, and creatine kinase-myocardial band. In addition to up-regulating Bcl-2 and downregulating Bax, and inhibiting Caspase 3 activity in the rat myocardium, ADMSCs-ex concurrently significantly reduced I/R-induced myocardial apoptosis. In addition, Wnt3a, p-GSK-3β (Ser9), and -catenin expression were not as strongly inhibited by I/R and H/R, which allowed ADMSCs-ex to induce the activation of Wnt/β-catenin signaling (72) more clearly. *In vitro*, exosomes from MSCs were able to reduce the production of ROS and cell apoptosis in H9C2s (Liu et al., 2017[66]). Similarly, *in vivo*, exosome injections significantly decreased apoptosis and the size of the myocardial infarct, increased myocardial LC3B expression, and improved heart function in rats that had undergone I/R injury. In fact, by triggering cardiomyocyte autophagy via the AMPK/mTOR and Akt/mTOR pathways, MSC-derived exosomes could lower MI (Liu et al., 2017[66]).

Given that microRNA-132 (miR-132) controls endothelial cell behavior during angiogenesis and that delivering microRNAs safely and effectively *in vivo* is uncommon, ischemic diseases may benefit from developing an ideal vehicle for miR-132 delivery (Ma et al., 2018[75]). MiR-132 can be shown through exosomes made from MSCs to treat myocardial ischemia. In HUVECs pretreated with exosomes, the expression of the miR-132 target gene RASA1 was inversely correlated with that of miR-132, demonstrating that RASA1 was a direct target of miR-132. Endothelial cells formed more tubes when exosomes carrying miR-132 were used as a method of miRNA transfer (Ma et al., 2018[75]). Additionally, subcutaneous injection of HUVECs pretreated with miR-132 exosomes in nude mice significantly improved their *in vivo* angiogenesis capacity. Furthermore, the transplantation of miR-132 exosomes in mice with ischemic hearts significantly increased the neovascularization in the peri-infarct zone while maintaining heart functions (Ma et al., 2018[75]). The miR-210-enriched MSCs-exosome induces both *in vitro* and *in vivo* pro-angiogenic effects. In HUVECs, MSC-exosome treatment decreased the expression of the miR-210 target gene Efna3, which prevents angiogenesis (Wang et al., 2017[124]). MSC-exosomes are satisfactory in enhancing angiogenesis and exert therapeutic effects on MI; their pro-angiogenic effect may be related to a miR-210-Efna3-reliant mechanism (Wang et al., 2017[124]). Additional studies have shown that exosomes from ADSCs overexpressing SIRT1 (ADSCs-SIRT1-Exos) increased the expression of C-X-C motif chemokine 12 (CXCL12) and nuclear factor E2 related factor 2 (Nrf2) in AMI-EPCs, which promoted migration and tube formation of AMI-EPCs (Huang et al., 2020[49]). ADSCs-SIRT1-exosome treatment increased survival, aided myocardial function recovery, decreased infarct size, and prevented post-AMI left ventricular remodeling. It also inspired vasculogenesis and reduced AMI-related myocardial inflammation. Thus, ADSCs-SIRT1-exosome may attract EPCs to the repair site, and this attraction may be aided by Nrf2/CXCL12/CXCR7 signaling (Huang et al., 2020[49]).

It has been demonstrated that MSC-derived exosomes play a role in macrophage immunomodulation following myocardial ischemia/reperfusion (I/R) and promoting angiogenesis. The polarization of M1 macrophages to M2 macrophages in animal models of MI was altered by MSC-Exo administration (Zhao et al., 2019[137]). MiR-182 was identified as a potential candidate mediator of macrophage polarization by miRNA sequencing of MSC-exosome and bioinformatics analysis, with toll-like receptor 4 (TLR4) identified as a downstream target. MiR-182's influence on macrophage polarization was somewhat attenuated in MSC-Exo when it was reduced. In a mouse model of myocardial I/R, knockdown of TLR4 also provided cardioprotective efficacy and decreased inflammation level (Zhao et al., 2019[137]).

Similarly, LPS preconditioning BMSC-derived exosomes may be a fruitful cell-free treatment plan for managing MI. Exosomes from BMSCs increased M2 macrophage polarization while decreasing M1 macrophage polarization in response to LPS stimulation (Xu et al., 2019[130]), inhibiting the LPS-dependent NF-κB signaling pathway and partially activating the AKT1/AKT2 signaling pathway (Xu et al., 2019[130]). Further, intramyocardial injection of MSC-EXO enriched in miRNA-181a in a mouse model of myocardial I/R injury resulted in significant protection against various immune-related genes via the miRNA-mRNA network. MiRNA-181a delivery by MSC-exosome combined the immune-suppressing properties of miRNA-181a and the cell targeting capabilities of MSC-exosome to have a more substantial therapeutic effect on myocardium I/R injury (Wei et al., 2019[127]). Another study used ADSC-exosome therapy to lessen the severity of MI-induced cardiac damage by preventing cardiac dysfunction, cardiac apoptosis, cardiac fibrosis, and inflammatory responses both *in vitro* and *in vivo*. The ADSC-exosome treatment additionally supported macrophage M2 polarization (Deng et al., 2019[29]). Further research revealed that the ADSC-exosome-mediated myocardial repair was mediated by S1P/SK1/ S1PR1 signaling. The downregulation of S1PR1 under hypoxic conditions, which increased the expression of NF-κB and TGF-1, and reduced the fibrosis and inflammatory response brought on by MI, also reversed the ADSC-exosome-induced macrophage M2 polarization (Deng et al., 2019[29]). A study of the effectiveness of MSC exosomes in a porcine model of myocardial infarction (MI) showed apparent effects of systemic exosomes administered over 7 days to decrease infarct size with largely unaltered cardiac function (Charles et al., 2020[18]). The infarct size is significantly reduced (30-40 %) after 7 days of IV exosome administration, as measured at 7 and 28 days after MI. Additionally, exosome therapy decreased transmural and weakened wall thinning in the infarct zone. Pigs treated with exosomes demonstrated a largely sustained level of LV function and marked improvement in falls in fractional wall thickening (Charles et al., 2020[18]). Additionally, MSCs-Exo-treated with IFN have more substantial cardioprotective effects in MI. IFNγ-exosome accelerated H9c2 migration and the development of tube-like structures while halting OGD-induced apoptosis (Zhang et al., 2022[136]). Comparatively to Ctrl-exosome treatment, IFNγ-Exo treatment decreased cardiomyocyte apoptosis, reduced fibrosis, and enhanced cardiac function. In IFNγ-primed MSCs, MiR-21 was markedly up-regulated and suppressed the expression of BTG anti-proliferation factor 2 (BTG2). Under OGD conditions, BTG2 induced apoptosis in H9c2 cells and blocked the protective effects of miR-21 (Zhang et al., 2022[136]). In line with this, miR-25-3p levels in cardiomyocytes significantly increased due to exosome uptake (Zhang et al., 2022[136]). Exosomal miR-25-3p from MSCs ameliorated MI by targeting pro-apoptotic proteins, as demonstrated by the mechanistically demonstrated direct targeting of the pro-apoptotic genes FASL and PTEN and subsequent reduction in their protein levels (Zhang et al., 2022[136]).

Furthermore, Exo secreted by MSCs undergoing hypoxia conditioning was discovered to have anti-ischemic properties. Significant enrichment of miR-125b-5p was found in Hypo-Exo, according to the miRNA array (Zhu et al., 2018[139]). This study provides evidence for a novel mechanism whereby the miR125b-5p produced by Hypo-Exo promotes cardiomyocyte apoptosis and ischemic cardiac repair. Exosomes from HIF-1-modified MSCs also restored the impaired angiogenic capacity, migratory function, and proliferation of hypoxia-damaged HUVECs (Sun et al., 2020[117]). In the rat MI model, HIF-1-overexpressed exosomes simultaneously preserved heart function by encouraging neovessel development and preventing fibrosis (Sun et al., 2020[117]). Exosomes produced by MSCs that overexpress SDF1 (Gong et al., 2019[40]) and CXCR4 (Kang et al., 2015[56]) also helped to improve cardiac remodeling, reduce infarct size, and promote angiogenesis, all of which helped to restore cardiac function; this was accomplished primarily by activating the PI3K/AKT axis and preventing autophagy in ischemic myocardial cells.

See also Table 3(Tab. 3) (References in Table 3: Chen et al., 2017[20]; Cheng et al., 2020[21]; Feng et al., 2014[36]; Huang et al., 2020[49][51][52]; Lai et al., 2020[61]; Li et al., 2020[62]; Luo et al., 2017[73]; Ma et al., 2017[74], 2018[75][76]; Ni et al., 2019[92]; Pan et al., 2019[95]; Peng et al., 2020[96]; Shao et al., 2017[112]; Wang et al., 2017[123][124]; Wei et al., 2019[127]; Xiao et al., 2018[129]; Yu et al., 2015[134]; Zhao et al., 2019[137]; Zhu et al., 2018[139], 2021[140]).

## Detection of Cardiac Biomarkers in Exosomes

Specific cardiac biomarkers may be present in exosomes derived from cardiac cells or found in the bloodstream, and these biomarkers can reveal important details about cardiac health and disease (Emanueli et al., 2016[31]). Identifying cardiac biomarkers in exosomes may provide information about the pathological processes in the heart and even function as a non-invasive diagnostic or prognostic tool (Lu et al., 2019[71]).

Troponins are proteins that control the contraction of the cardiac muscle. Cardiovascular troponins with elevated levels, notably troponin T (cTnT) and troponin I (cTnI), are used to diagnose myocardial infarction and are diagnostic markers for myocardial injury (Liu et al., 2023[67]). Exosomes made from damaged cardiomyocytes can be found to contain cardiac troponins. The heart responds to increased pressure or volume overload by secreting natriuretic peptides like B-type natriuretic peptide (BNP) and N-terminal pro-B-type natriuretic peptide (NT-proBNP) (Cao et al., 2019[15]). They act as biomarkers for identifying and tracking heart failure. Exosomes derived from cardiac cells and circulating exosomes have both been found to contain natriuretic peptides and their receptors. Additionally, myosin-binding protein C (cMyBP-C) plays a crucial regulatory role in cardiac muscle contraction (Harris, 2019[47]). Hypertrophic cardiomyopathy is connected to mutations in the MYBPC3 gene that codes for cMyBP-C. Exosomes from patients with hypertrophic cardiomyopathy have been found to contain cMyBP-C, indicating the possibility that this molecule could serve as a biomarker for the condition (Gao et al., 2022[38]).

Small non-coding RNA molecules called microRNAs (miRNAs) control the expression of genes. Numerous miRNAs are biomarkers for cardiac disorders, and exosomes can contain them. Myocardial infarction and heart failure are linked, for instance, to miR-208a, miR-133a, and miR-1 (Bostjancic et al., 2010[13]). The identification of these miRNAs in exosomes may shed light on cardiac pathologies. Exosomes may contain biomarkers linked to cardiac ischemic circumstances. Exosomes from ischemic myocardium, for example, may include the miRNAs miR-1, miR-133a, and miR-208a, which are linked to ischemic injury and cardiac damage (Bostjancic et al., 2010[13]). These exosome biomarkers can indicate the degree and scope of cardiac ischemia. Exosomes can also carry inflammatory markers linked to cardiac inflammation. Inflammatory biomarkers have been found in the exosomes of cardiac patients, including C-reactive protein (CRP), interleukins (like IL-6), and tumor necrosis factor-alpha (TNF-ɑ) (Albar et al., 2022[4]; Soeki and Sata, 2016[115]). These markers can help diagnose and follow up inflammatory cardiac conditions because they show the presence of cardiac inflammation.

## Clinical Trials Exploring MSC-Exosome Therapies for Cardiac Conditions

The potential of exosome therapies for different cardiac conditions is currently being studied in several clinical trials. The use of MSCs-exosome for treating patients with heart failure with preserved ejection fraction (HFpEF) is being investigated as part of the DREAM-HF phase II clinical trial (Bolli and Tang, 2022[12]). The trial's objectives are to evaluate the safety and effectiveness of MSC-exosome in enhancing exercise capacity, cardiac function, and quality of life (Bolli and Tang, 2022[12]). Exosomes derived from allogeneic BM-MSCs are being investigated for treating ST-elevation myocardial infarction (STEMI) patients in the PRECISE phase I/II clinical trial (Sanz-Ruiz et al., 2010[109]).

Exosomes derived from MSCs will be tested to see how they affect tissue repair and cardiac function. Similarly, the safety and effectiveness of delivering exosomes from BM-MNCs intracoronary to patients with AMI are being studied in the EVOMEND phase I/II clinical trial (Attar et al., 2021[6]). Exosomes made from autologous adipose tissue-derived (AD)-MSCs are also being administered intravenously to patients with acute AMI as part of the EXOSOMA phase I clinical trial, which evaluates their safety and viability (Attar et al., 2021[6]; Fan et al., 2010[33]). The trial assesses how AD-MSC exosomes affect cardiac function and tissue repair.

These are just a few active clinical trials examining exosome therapies for cardiac conditions. These studies seek to shed light on the efficacy, safety, and viability of exosome-based treatments in enhancing cardiac function, fostering tissue regeneration, and possibly revolutionizing the management of various cardiac diseases. It is significant to note that the results of clinical trials are still pending, and additional study is required to determine the efficacy and long-term effects of exosome therapies in cardiac patients (Chou et al., 2014[23]).

## Challenges and Concerns

There is still a need for reliable exosome isolation and characterization techniques. Standard protocols must be established to guarantee isolated exosomes' reproducibility, purity, and quality (Rezaie et al., 2022[106]). Achieving high loading efficiency of therapeutic cargo into exosomes is still technically challenging. The best therapeutic results depend on the development of effective and dependable procedures for therapeutic agent loading into exosomes while maintaining their stability and functionality (Yamashita et al., 2018[131]). Exosome delivery and precisely targeting the desired cardiac cell types or tissues are difficult. It is necessary to conduct more research to strengthen targeting tactics, increase the specificity of exosome-cell interactions, and get past potential obstacles like the blood-brain barrier or the development of scar tissue. Exosome production at a larger scale for medical uses presents a challenge (Mehryab et al., 2020[84]). Scalable manufacturing processes must be created to produce therapeutic-grade exosomes in sufficient quantities and with reliable quality and effectiveness.

Further research is needed to determine the durability and safety of exosome-based therapies over the long term (Adamiak and Sahoo, 2018[1]). It is crucial to comprehend potential adverse effects, immune responses, and the determination of therapeutic possessions over time to guarantee the long-term advantages and security of exosome-based treatments (Ahmadi and Rezaie, 2021[2]; Ludwig et al., 2019[72]). Exosome-based therapies are still in their infancy, and regulatory frameworks for their clinical translation and approval are changing. Exosome-based therapies must be successfully translated into clinical practice, which will require overcoming regulatory obstacles and establishing suitable guidelines and standards (Cheng et al., 2017[22]).

Exosome therapies can be expensive, so it's essential to consider their accessibility to all patients, including those from low socioeconomic statuses. Fair resource allocation, prioritizing patients based on clinical need, and preventing disparities in entrance to potentially helpful treatments are all ethical considerations (Ayala‐Mar et al., 2019[7]). Careful thought must be given to exosome therapy's security and long-term effects. While exosomes derived from autologous sources may have less immunogenicity, potential risks, such as immune reactions, off-target effects, or unexpected long-term consequences, must be carefully evaluated through rigorous pre-clinical and clinical studies (Ding et al., 2021[30]). Monitoring patients receiving exosome therapy and long-term follow-up is crucial to assess the safety and guarantee patient well-being. Exosome therapies develop, and regulatory oversight ethics become increasingly important. Exosome-based treatments must be developed, produced, and used in clinical settings following ethical standards, maintaining patient safety, and adhering to pertinent legal and regulatory frameworks, requiring adequate regulation and oversight (Rezaie et al., 2022[106]).

## Conclusion

Exosomes have the potential to significantly improve cardiac patient outcomes and play a substantial role in cardiac health. Exosomes can promote the growth and differentiation of cardiac progenitor cells, which produces new, functional cardiomyocytes. Exosomes are a promising therapeutic option for treating the adult heart's constrained capacity for regeneration because of their regenerative potential. Specific molecules, such as proteins, RNA, and miRNA, are found in exosomes derived from cardiac cells and can be used as diagnostic biomarkers for cardiac diseases. Exosomal biomarker analysis enables non-invasive detection, monitoring, and risk stratification of cardiac conditions, offering important visions into disease progression and treatment response. Exosomes can be used as organic nanocarriers for the delivery of specific drugs. They can transport various therapeutic molecules to particular cardiac cells or tissues by encasing and delivering them as proteins, nucleic acids, small molecules, or gene-editing agents. This targeted delivery method increases treatment effectiveness while reducing systemic toxicity and off-target effects.

Cardiologists can practice personalized medicine using autologous exosomes from a patient's cells. Exosome therapies can be customized to each patient, maximizing treatment efficacy and lowering immunogenicity by utilizing the patient's biological characteristics. Exosome therapies can open new avenues for cardiac regeneration, non-invasive diagnosis, targeted drug delivery, cardioprotection, and personalized medicine. Researchers and clinicians can address current therapies' shortcomings and potentially enhance patient outcomes by utilizing the therapeutic potential of exosomes. More research, clinical trials, and technological developments are required to understand the impact of exosome therapies in cardiology fully.

## Declaration

### Ethics approval and consent to participate

Not applicable.

### Consent for publication

Not applicable.

### Availability of data and materials

Not applicable.

### Competing interests

There is no conflict of interest.

### Funding

No funders.

## Figures and Tables

**Table 1:**
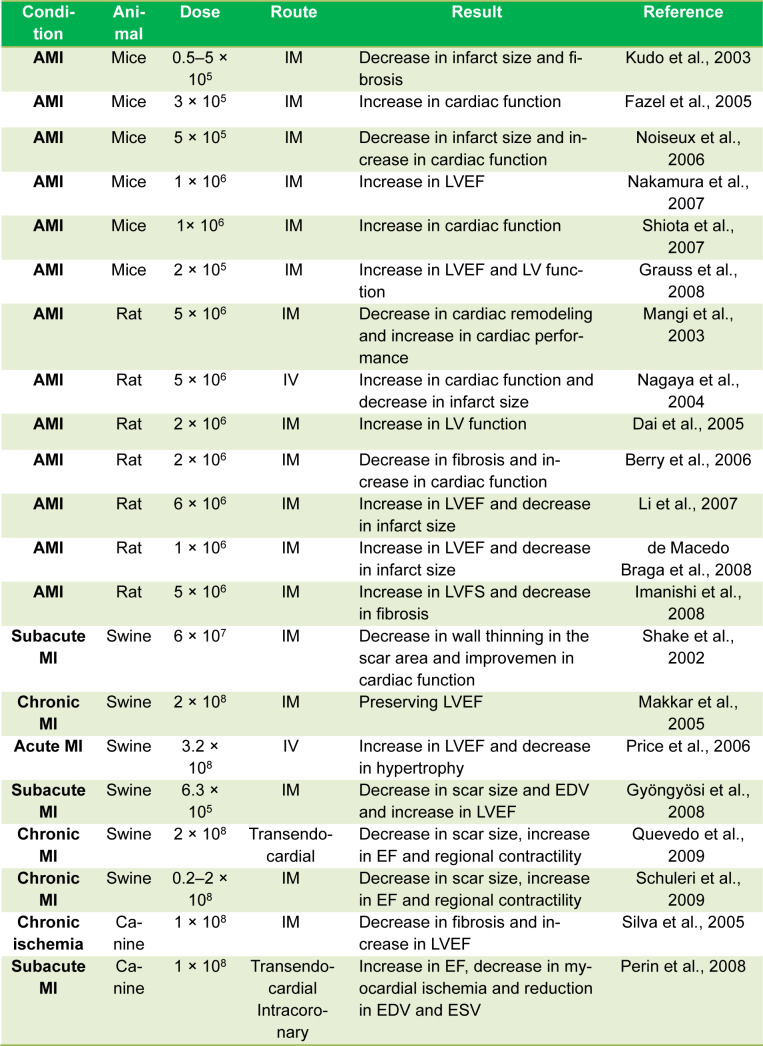
MSCs therapy in cardiac regeneration

**Table 2:**
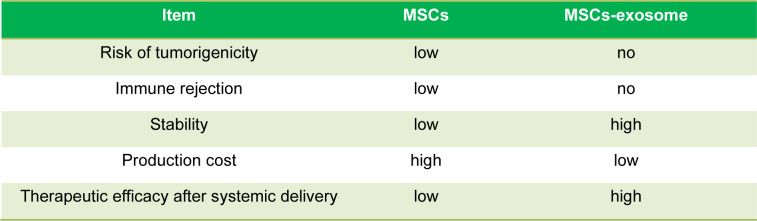
The advantages of MSC-derived exosomes over their parent cells (Hassanzadeh et al., 2021; Moghadasi et al., 2021)

**Table 3:**
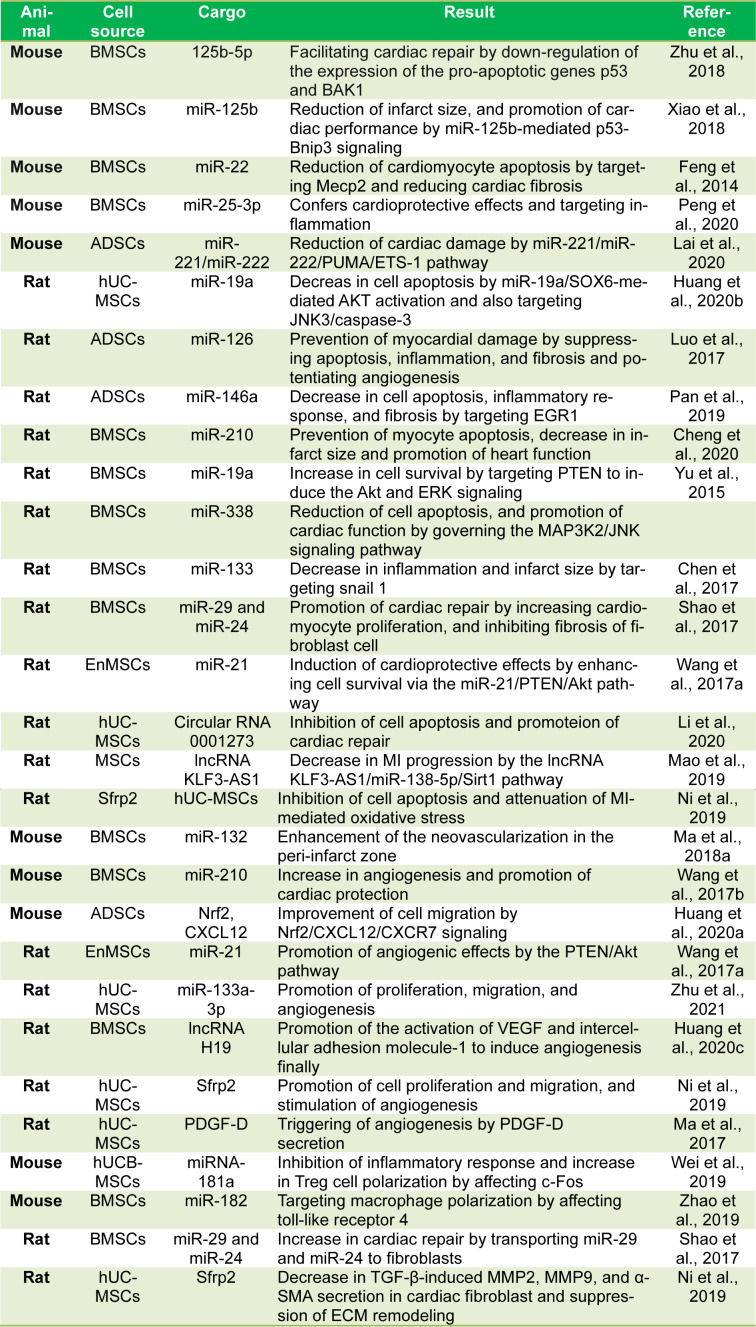
MSCs-exosome therapy in MI
